# A short D-enantiomeric antimicrobial peptide with potent immunomodulatory and antibiofilm activity against multidrug-resistant *Pseudomonas aeruginosa* and *Acinetobacter baumannii*

**DOI:** 10.1038/s41598-017-07440-0

**Published:** 2017-07-31

**Authors:** Mohamed F. Mohamed, Anna Brezden, Haroon Mohammad, Jean Chmielewski, Mohamed N. Seleem

**Affiliations:** 1 Department of Comparative Pathobiology, Purdue University, West Lafayette, IN 47907 USA; 2 Department of Chemistry, Purdue University, West Lafayette, IN 47907 USA; 3 Purdue Institute for Inflammation, Immunology, and Infectious Disease, Purdue University, West Lafayette, IN 47907 USA

## Abstract

Antimicrobial peptides (AMPs) represent a promising therapeutic alternative for the treatment of antibiotic-resistant bacterial infections. The present study investigates the antimicrobial activity of new, rationally-designed derivatives of a short α-helical peptide, RR. From the peptides designed, RR4 and its D-enantiomer, D-RR4, emerged as the most potent analogues with a more than 32-fold improvement in antimicrobial activity observed against multidrug-resistant strains of *Pseudomonas aeruginosa and Acinetobacter baumannii*. Remarkably, D-RR4 demonstrated potent activity against colistin-resistant strains of *P. aeruginosa* (isolated from cystic fibrosis patients) indicating a potential therapeutic advantage of this peptide over several AMPs. In contrast to many natural AMPs, D-RR4 retained its activity under challenging physiological conditions (high salts, serum, and acidic pH). Furthermore, D-RR4 was more capable of disrupting *P. aeruginosa* and *A. baumannii* biofilms when compared to conventional antibiotics. Of note, D-RR4 was able to bind to lipopolysaccharide to reduce the endotoxin-induced proinflammatory cytokine response in macrophages. Finally, D-RR4 protected *Caenorhabditis elegans* from lethal infections of *P*. *aeruginosa* and *A*. *baumannii* and enhanced the activity of colistin *in vivo* against colistin-resistant *P*. *aeruginosa*.

## Introduction

The emergence and spread of multidrug-resistant strains of *Pseudomonas aeruginosa* and *Acinetobacter baumannii* represents a serious global medical threat^1–3^. This highlights the need for discovering new therapeutic agents and alternative approaches to treat these highly-challenging drug-resistant infections.

Antimicrobial peptides (AMPs) possess several unique features that support their utility as antibacterial agents. However, naturally-derived AMPs have been not been successfully translated for use clinically due to limitations that include moderate antimicrobial activity, toxicity to host tissues, intolerance to physiological conditions, sensitivity to enzymatic degradation, and high manufacturing costs due to their complex design^4–6^. Thus, successful translation of AMPs into the clinic requires these limitations to be overcome.

We recently identified a small synthetic peptide, RR (12 amino acids), that exhibited potent antibacterial activity against methicillin-resistant *Staphylococcus aureus* (MRSA) both *in vitro* and in a murine staphylococcal skin infection model^7, 8^. However, the antibacterial activity of RR against Gram-negative pathogens was found to be very weak. In the present study, derivatives of RR were designed in order to enhance the peptide’s potency and spectrum of antibacterial activity (against *P*. *aeruginosa* and *A*. *baumannii*) while also reducing toxicity to mammalian cells. The stability of the derivatives of RR to proteolytic degradation and inactivation by salts and serum was evaluated. Additionally, the antibacterial mechanism of action and immune-modulatory activity of the new peptides was explored. Furthermore, the peptides’ ability to eradicate preformed *P. aeruginosa* and *A. baumannii* biofilms and to clear macrophage cells infected with *P. aeruginosa* was examined. Finally, the antibacterial activity of the most promising analogue (D-RR4) was investigated *in vivo* in a *Caenorhabditis elegans* model of bacterial infection. Taken altogether, our findings indicate that D-RR4 is a promising AMP that warrants further investigation.

## Results and Discussion

### Design of new analogues of RR with improved spectrum of antibacterial activity

In order to rationally design more potent, less toxic analogues of RR, the following structural features were taken into consideration, (i) peptides were designed to maintain the amphipathic helices by converging the hydrophobic residues on one side and the hydrophilic residues on the other side of the helical axis as shown in the helical wheel diagram (Supplementary Fig. S1A); (ii) the hydrophobic face of certain peptides was disrupted via inclusion of a single hydrophilic residue (lysine or arginine) in order to decrease toxicity and improve selectivity toward bacteria^9, 10^; (iii) the length of amino acids was kept short (12–14 residues) in order to reduce cost of production and minimize host toxicity; (iv) amino acids known to play a crucial role in the antimicrobial activity of RR, such as tryptophan and arginine^11–13^, were maintained in all modified derivatives; (v) the cationic net charge of the designed analogues was increased by adding one or more polar amino acids, particularly lysine residues, to enhance antibacterial activity^14, 15^. The peptides were synthesized as described above and mass spectrometry was utilized to confirm the peptides’ molecular weight (Table 1).

**Table 1:** Amino acid sequence and physicochemical properties of peptides used in this study.

	Amino acid sequences	Length	Calculated molecular weight	Actual molecular weight	Charge	Hydrophobic amino acids	Mean hydrophobicity <H>	Hydrophobic moment<µH>
RR	WLRRIKAWLRR	11	1553.92	1553.91	+5	54%	0.453	0.831
RR1	WKRRIKIWKKIR	12	1711.17	1711.16	+7	41%	0.242	0.659
RR2	WIRRIKKWIRRVHK	14	1975.46	1975.45	+7	42%	0.303	0.901
RR3	WLRRIKAWLRRKRK	14	1966.45	1966.44	+8	42%	0.142	0.652
RR4	WLRRIKAWLRRIKA	14	1866.33	1866.32	+6	57%	0.436	0.759
D-RR4	wlrrikawlrrika-NH_2_^a^	14	1866.33	1866.32	+7	57%	0.436	0.759

^a^Small underlined residues represent D-amino acids.

The antibacterial activity of RR and its modified derivatives was next investigated against multidrug-resistant strains of *P. aeruginosa* and *A. baumannii* (Supplementary Tables S1, S2 and S3). RR displayed weak antibacterial activity against both *P. aeruginosa* and *A. baumannii* with MIC_50_ (minimum inhibitory concentration that inhibited 50% of all isolates tested) values equal to 64 µM and MIC_90_ (minimum inhibitory concentration that inhibited 90% of all isolates tested) values equal to 128 µM (Table 2). All modified derivatives, except RR1, demonstrated improved antibacterial activity compared to RR.

**Table 2:** Minimum inhibitory concentration (MIC) (µM) of peptides against clinical and drug-resistant isolates of *P. aeruginosa* and *A. baumannii*.

Strain	Designation	RR	RR1	RR2	RR3	RR4	D-RR4	LL-37	Indolicidin	Colistin
***P. aeruginosa***	PAO1	64	>256	4	8	4	2	16	64	0.5
	ATCC 9721	64	>256	8	16	8	4	32	>256	0.5
	ATCC BAA-1744	128	>256	16	16	8	8	32	64	0.5
	ATCC 27853	64	256	8	8	8	4	16	64	0.25
	ATCC 35032	64	>256	16	16	8	4	128	>256	0.5
	ATCC 25619	64	>256	8	4	8	4	16	>256	0.25
	ATCC 9027	64	>256	4	4	4	2	16	32	0.25
	ATCC 15442	128	>256	16	16	8	4	32	64	0.25
	ATCC 10145	128	>256	8	16	8	4	>256	128	0.5
***A. baumannii***	ATCC BAA-1605	32	>256	4	16	2	2	4	32	0.25
	ATCC 19606	16	>256	2	16	2	2	2	8	0.125
	ATCC BAA-747	64	>256	4	8	2	2	8	32	0.25
	NR-9667	32	>256	4	32	4	4	4	32	0.0625
	NR-13374	32	128	4	8	4	4	4	8	0.125
	NR-13375	32	>256	2	8	2	2	4	32	0.125
	NR-13382	32	>256	4	16	4	8	4	32	0.5
	NR-17777	16	>256	4	8	2	4	4	32	0.5
	NR-17778	16	>256	4	8	2	2	4	32	0.0625
	NR-17780	32	>256	4	8	2	2	4	32	0.25
	NR-17783	32	>256	4	8	4	4	4	16	0.0625
	NR-17784	64	>256	4	4	2	2	4	64	0.0625
	NR-17785	32	>256	4	8	2	2	4	32	0.25
	NR-17786	16	>256	4	4	2	2	4	16	0.0625
	NR-19298	32	>256	2	4	2	2	2	8	0.25
	NR-19299	64	>256	4	32	4	4	8	64	0.25
**MIC**_**50**_^**a**^		**64**	>**256**	**4**	**8**	**4**	**2**	**4**	**32**	**0.25**
**MIC**_**90**_^**b**^		**128**	>**256**	**8**	**16**	**8**	**4**	**32**	**128**	**0.5**

^a^MIC_50_ (The minimum inhibitory concentration that inhibited growth of 50% of all isolates).

^b^MIC_90_ (The minimum inhibitory concentration that inhibited growth of 90% of all isolates).

Substitution of the hydrophobic residues (leucine) in RR with cationic residues (lysine) (to generate RR1) increased the cationic charge from +5 (RR) to +7 (RR1) and decreased the hydrophobicity from 0.45 to 0.24. As a result, RR1 was found to be inactive against all tested bacterial strains (MIC > 256 µM). This most likely is due to the inability of RR1 to form an alpha helix, as observed in the presence of membrane-mimicking environments (Supplementary Fig. S1B), due to disruption of the peptide’s amphipathicity (Supplementary Fig. S1A). These findings highlight that hydrophobicity plays a more critical role in the antibacterial activity of helical AMPs moreso than increasing the number of cationic charges. Indeed, the peptide with the highest helical content that maintained amphipathicity (RR4) was found to have the most potent antibacterial activity. RR4, which also had an increased cationic charge, hydrophobicity and amphipathicity as compared to RR, exhibited very potent activity against both *P. aeruginosa* and *A. baumannii* with MIC_50_ and MIC_90_ values equal to 4 and 8 µM. This represents a 16-fold improvement in the MIC over RR. Replacing a lysine residue in RR4 with a histidine residue and substitution of two leucine residues with isoleucine resulted in RR2. RR2 was also more potent than RR (MIC_50_ value of RR2 was equal to 4 µM and its MIC_90_ value was equal to 8 µM).

Susceptibility of peptides to digestion by proteases serves as a major impediment to the clinical application of peptides^16^. In order to overcome this limitation, we designed a D-enantiomer of the most active peptide, RR4. D-RR4 was amidated at the C-terminus in order to increase its cationic net charge. The MIC of D-RR4 ranged from 2 to 8 µM with MIC_50_ and MIC_90_ values equal to 2 and 4 µM, respectively. The antimicrobial activity of D-RR4 was one-fold more potent than RR4 against most *P. aeruginosa* strains. However, D-RR4 was similar to RR4 against most *A. baumannii* strains and one-fold less potent against two strains (NR-13382 and NR-17777) (Table 2). The reason for this minor discrepancy in antimicrobial activity between RR4 and D-RR4 against different bacterial isolates is not clear. Previously it was reported that membrane-targeting antimicrobial peptides work without the need for receptors and therefore D isomers have similar antimicrobial activity to L isomers^17^. However, there are several studies that have reported that D isomers demonstrated different antimicrobial responses than their L isomer counterparts against different bacterial isolates. This may be due to the fact that the backbone of D and L isomers of helical AMPs, in three-dimensional space, have opposite rotations and therefore their side chains will have differing topologies^18^. A possible alternative explanation for the difference in antimicrobial activity between RR4 and D-RR4 is that *P. aeruginosa and A. baumannii* produce differing amounts of proteases^19–21^. *P. aeruginosa* secretes two major proteases, *Pseudomonas* elastase and an alkaline protease^22^. However, *A. baumannii* scarcely produces proteases^19–21^. Thus, the higher levels of proteases secreted by P. aeruginosa may make it less sensitive to the effect of the peptides.

### Circular dichroism spectroscopy (CD)

The secondary structure of designed peptides in aqueous and membrane mimicking solvents^11, 14^, such as TFE and SDS, was investigated using CD spectroscopy. In all, the peptides demonstrated a mostly random coil conformation in the aqueous solvent as demonstrated by a broad minimum peak at 200 nm. The starting peptide, RR, was found to have an increase in helical content in the presence of membrane-mimicking solvents (TFE and SDS) up to 20% and 12%, respectively (as determined by the mean residue ellipticity at 222 nm, Supplementary Fig. S1B). All of the modified peptides, with the exception of the non-amphipathic RR1, changed their conformation to some extent in the presence of membrane-mimicking solvents (Supplementary Fig. S1B). Peptide RR2, which contained no leucine residues in the hydrophobic face compared to RR, displayed very modest changes in the CD spectra in the presence of membrane-mimicking solvents. The pronounced maximum at 190 nm and strong minima at 208 and 222 nm displayed by RR3 and RR4, however, are highly indicative of alpha helicity. RR4 was found to be somewhat more helical than RR, with the addition of SDS and TFE, with helical contents of about 30%. In this study, an all D-amino acid version of the RR4 peptide (D-RR4) was prepared with an amidated C-terminus. As expected, the CD spectra of D-RR4 was the mirror image of the RR4 peptide, with mean residue ellipticities that were opposite in sign, but somewhat higher than RR4 with the addition of SDS and TFE (about 40% helical content) due to the addition of the C-terminal amide (Supplementary Fig. S1B).

### RR analogues display potent antibacterial activity against colistin-resistant *P. aeruginosa*

After confirming the potent antibacterial activity of the RR analogues against colistin-sensitive *P. aeruginosa* isolates, we moved to examine if the peptides’ activity could be retained against a panel of clinical isolates of colistin-resistant *P. aeruginosa* (isolated from cystic fibrosis patients)^23^. Although these isolates showed high resistance to colistin and several natural peptides, our designed peptides retained their potent antibacterial activity (Table 3). The most potent peptide against colistin-resistant *P. aeruginosa* isolates was D-RR4 with an MIC_50_ value of 4 µM. This represents a 32-fold enhancement in the antimicrobial activity of this peptide compared to RR (MIC_50_ 128 µM). RR2 demonstrated improved activity over RR with a MIC_50_ value equal to 32 µM. RR3 and RR4 were found to be more potent than RR2 with MIC_50_ values equal to 16 and 8 µM, respectively. However, the antimicrobial activity of indolicidin was abolished against all tested isolates. LL-37 showed weak to moderate activity against some strains (MIC_50_ of 64 µM), while no activity was observed against five colistin-resistant strains (Table 3).

**Table 3:** Minimum inhibitory concentration (MIC) (µM) of peptides against clinical isolates of colistin-resistant *P. aeruginosa* isolated from cystic fibrosis patients.

Sstrain	RR	RR1	RR2	RR3	RR4	D-RR4	LL-37	Indolicidin	Colistin
1109	128	>128	16	8	4	2	64	>128	128
1125	128	>128	32	32	8	4	128	>128	32
1571	128	>128	32	8	4	2	16	>128	256
1603	128	>128	16	8	8	4	32	>128	64
16011	>128	>128	32	32	16	4	>128	>128	128
1017	>128	>128	16	64	32	16	>128	>128	>256
1020	128	>128	16	8	4	2	32	>128	256
1133	64	>128	32	32	16	8	32	>128	32
1015	>128	>128	16	8	32	4	>128	>128	>256
1016	>128	>128	32	32	32	16	>128	>128	>256
1131	128	>128	32	16	8	4	16	>128	64
**MIC**_**50**_	**128**	**>128**	**32**	**16**	**8**	**4**	**64**	**>128**	**128**
**MIC**_**90**_	**>128**	**>128**	**32**	**32**	**32**	**16**	**>128**	**>128**	**>256**

### D-RR4 is resistant to proteolytic digestion

To confirm the stability of D-RR4 to proteolytic digestion, we evaluated its antimicrobial activity after treatment with mammalian (trypsin) and bacterial (proteinase K) proteases for four hours. As presented in Table 4, the antibacterial activity of the L isoform of RR4 was completely abolished in the presence of both trypsin and proteinase K. However, the MIC of the D isoform of RR4 (D-RR4) was not sensitive to proteolytic degradation when tested against *P. aeruginosa*.

**Table 4:** Minimum inhibitory concentration values of designed peptides in the presence of salts, pH 5.5 or pH 6.5, fetal bovine serum (FBS, 2%, 5% or 10%), human serum (HS, 2% or 5%), and plasma from human donors (2% or 5%) against *P. aeruginosa* PAO1.

	Control	NaCl 100 mM	NaCl 150 mM	CaCl2 8 µm	MgCl2 1 mM	pH 5.5	pH 6.5	FBS 2%	FBS 5%	FBS 10%	HS 2%	HS 5%	H plasma 2%	H plasma 5%	Trypsin 1:500	Proteinase K 1:500
RR	64	64	128	64	256	128	128	64	64	128	256	256	128	256	ND^a^	ND
RR2	4	4	8	4	16	8	4	8	32	128	128	128	8	16	ND	ND
RR3	8	16	16	8	32	32	16	16	32	64	32	128	16	16	ND	ND
RR4	4	4	8	4	16	8	2	4	4	16	16	32	16	32	>64	>64
D-RR4	2	2	4	2	8	4	1	2	2	4	2	4	2	2	2	2

^a^ND, not determined.

We confirmed the stability of D-RR4 to proteases utilizing HPLC. As demonstrated in Supplementary Fig. S2, incubation of the L isoform of RR4 with proteases (for four hours) led to the degradation of the intact peptide, resulting in multiple lower molecular weight peaks, hence explaining the loss of antimicrobial activity observed earlier (Table 4). However, D-RR4 remained intact after protease treatment, even after 24 hours of incubation (Supplementary Fig. S2).

### D-RR4 retains its potent antibacterial activity in the presence of salts, serum and acidic pH

A significant drawback of many AMPs is their antibacterial activity is impaired/lost in the presence of biological fluids (serum, plasma, high concentration of salts and acidic pH) which severely limits their clinical utility^4, 5^. With this point in mind, we assessed the antibacterial activity of our peptides against *P. aeruginosa* PAO1 under several biologically-relevant conditions. Overall, all designed peptides demonstrated low salt sensitivity (Table 4). All peptides exhibited a one-fold increase in the presence of 150 mM NaCl. In the epithelial cell secretions of a CF patients, the NaCl concentration is approximately 120 mM^6^; at this concentration all RR derivatives, particularly RR2, RR4 and D-RR4, maintained good antibacterial activity. Moreover, all peptides were not affected by CaCl_2_ concentration. However, in the presence of MgCl_2,_ there was a two- to four-fold decrease in the peptides’ antimicrobial activity (Table 4).

Recently, several studies reported that abnormally reduced pH in the airway surface liquid of CF patients (pH 6.8) inhibits the antimicrobial activity of host defense peptides and may impair the host’s immune response to pathogens^24, 25^. Therefore, we assessed the effect of acidity on the antibacterial activity of our designed peptides by determining their MIC at different pH (5.5 and 6.5). There was no significant difference in the MICs of most peptides at acidic pH (a one-fold decrease in MIC was observed at pH 5.5, except for RR3 where a four-fold decrease was observed).

In addition to salts and acidity, serum and plasma can also impair the antibacterial activity of conventional antibiotics and peptides either via proteolytic digestion or binding to protein or lipid fractions^26^. We thus moved to examine whether our peptides would exhibit diminished activity in the presence of serum or plasma. As presented in Table 4, most peptides were inactive in the presence of fetal bovine serum, human serum, and human plasma. Interestingly, D-RR4, retained its potent anti-*Pseudomonas* activity under the same test conditions.

### RR analogues exhibit an improved safety profile to mammalian cells

A significant limitation for translating AMPs into clinical applications is their toxicity to host (mammalian) cells^27^. Herein, we evaluated the cytotoxic effects of our peptide constructs against murine macrophages (J774.1A) and human keratinocytes (HaCaT cells), via the MTS assay. The half maximal effective concentration (EC_50_) of RR, RR1, RR2, RR3, RR4 and D-RR4 was 128/256, >256/>256, 128/64, 128/128, 64/128 and 64/64 µM, respectively, in the case of J774.1A/HaCaT cells (Supplementary Table S4). Supplementary Table S4 also presents the therapeutic index (TI) of the peptide constructs. All modified derivatives displayed a higher safety window than the parent peptide, RR.

Next, we assessed the hemolytic activity of the peptides on human red blood cells (RBCs). As presented in Supplementary Table S4, the HC_50_ value (the peptide concentration that resulted in 50% hemolysis of RBCs) for all peptides was equal to or exceeded 256 µM.

### RR4 and D-RR4 are potent disruptors of bacterial biofilms

**Figure 1:**
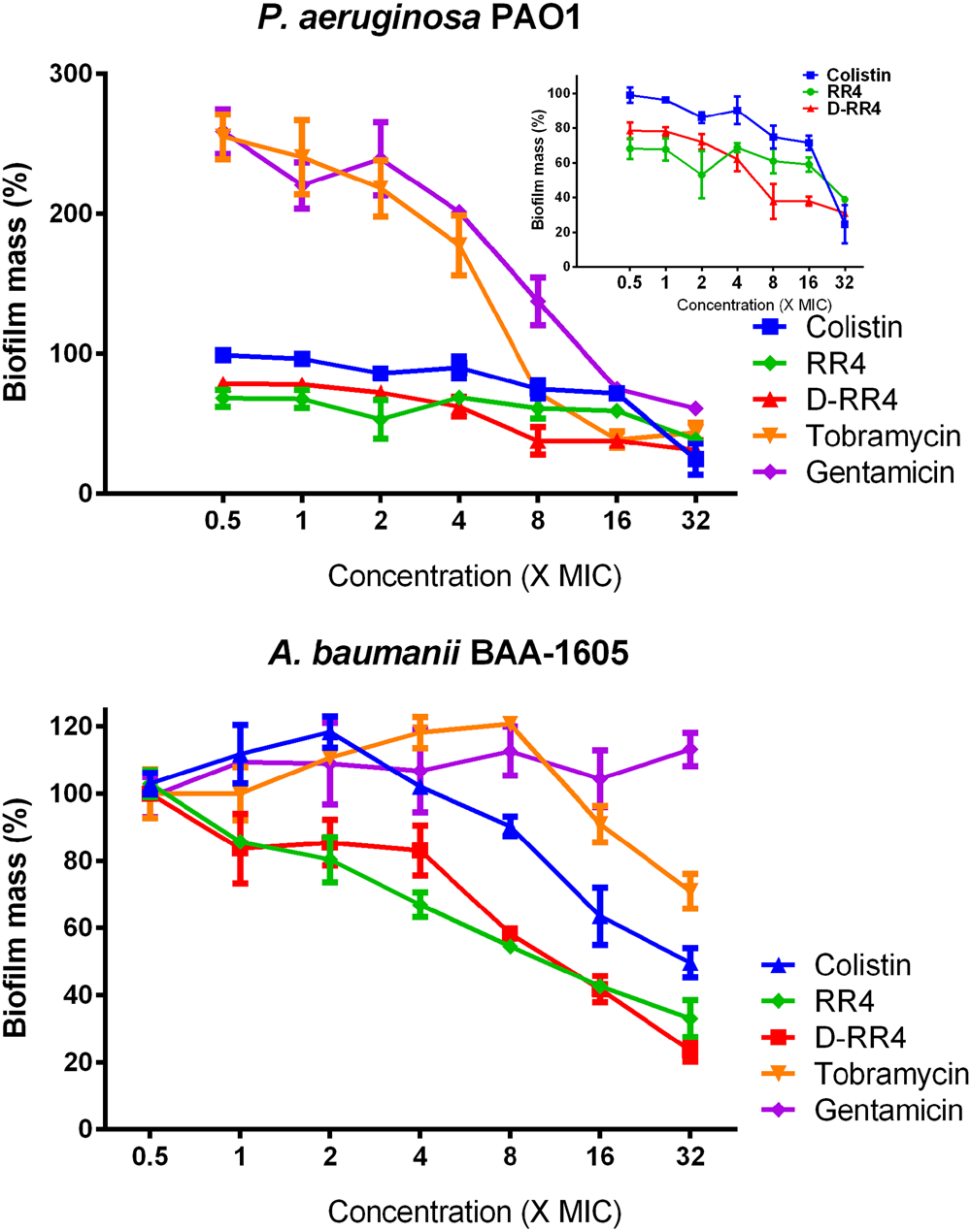
Efficacy of peptides on established biofilms of *P. aeruginosa* PAO1 and *A. baumannii* ATCC BAA-1605. Mature (24 hour) biofilms were treated with different concentrations of RR4, D-RR4, colistin, tobramycin and gentamicin for 24 hours at 37 °C. After incubation, wells were washed and biofilms were stained with 0.5% (w/v) crystal violet for 30 minutes. The dye was solubilized with ethanol (95%) and the optical density (595 nm) of the biofilm mass was measured. Experiments were repeated twice independently and the average values are reported.

We examined the capability of the most promising RR peptide analogues, RR4 and D-RR4, and control antibiotics to disrupt established biofilms of *P. aeruginosa* and *A. baumannii*. As shown in Fig. 1, RR4 and D-RR4 disrupted mature (24 hour) biofilms more significantly than several antibiotics. RR4 and D-RR4 demonstrated concentration-dependent biofilm-disrupting activity. RR4, at 4, 8, 16 and 32 × MIC, disrupted more than 31%, 39%, 41% and 61% of biofilm mass, respectively (*p* < 0.05). D-RR4 disrupted more than 30%, 40%, 60% and 75% of biofilm mass, respectively (*p* < 0.05), at the same concentrations. Colistin reduced more than 10%, 25%, 28%, and 75% of biofilm mass, respectively (*p* < 0.05), at the same concentrations. In comparison, gentamicin and tobramycin required a concentration equal to 32 × MIC to disrupt 50% of biofilm mass (*p* < 0.05).

Remarkably, RR4 and D-RR4 were capable of reducing 20–35% of *P. aeruginosa* biofilm mass at concentrations equivalent to 0.5× to 1 × MIC, respectively. In contrast, low concentrations of gentamicin and tobramycin enhanced biofilm production (100 to 240% increase compared to untreated samples), which is in agreement with previous findings^28^. For example, Hoffman *et al*. found that subinhibitory concentrations of tobramycin and gentamicin induced *P. aeruginosa* PAO1 biofilm formation^28^.

When RR4 and D-RR4 were examined against *A. baumannii* biofilm, a concentration-dependent activity was also observed (Fig. 1). Collectively, the data demonstrate that peptides RR4 and D-RR4 are more effective than conventional antibiotics in penetrating and disrupting adherent *P. aeruginosa* and *A. baumannii* biofilms.

### Time-kill assay against bacteria either in logarithmic or stationary phase of growth

After confirming that the AMPs possessed excellent antimicrobial activity against multidrug-resistant clinical isolates of *P. aeruginosa* and *A. baumannii*, we assessed the killing kinetics of RR and the most promising analogue, D-RR4 against *P. aeruginosa* PAO1 and *A. baumannii* ATCC BAA-1605 in logarithmic phase of growth. D-RR4 demonstrated rapid, concentration-dependent killing of both bacterial species (Supplementary Fig. 3). AMPs with fast bactericidal activity have several advantages including limiting the spread of infection, improving treatment prognosis, limiting the emergence of resistance, and potentially reducing the duration of treatment^29^.

Next, we examined the impact of RR and D-RR4 and select antibiotics against *P. aeruginosa* and *A. baumannii* in stationary-phase of growth. Treatment of stationary-phase *P. aeruginosa* PAO1 with 10 × MIC D-RR4 resulted in rapid bactericidal activity (three-log_10_ reduction) after three hours (Supplementary Fig. 3). Conventional antibiotics had minimal impact on stationary-phase *P. aeruginosa* PAO1. Complete eradication of stationary-phase *A. baumannii* ATCC BAA-1605 was achieved after treatment with D-RR4 for three hours (Supplementary Fig. 3). The superior activity of D-RR4, when compared to conventional antibiotics, against bacteria in stationary-phase of growth can be explained by its unique antimicrobial mechanism of action. Many antibiotics require bacteria to be growing and metabolically active in order to inhibit their molecular targets, such as protein synthesis (for tobramycin and gentamicin)^30^. In contrast, as elaborated further below, the positive charge present in D-RR4 serves as a point of attraction with negatively-charged bacterial cell membranes and consequently leads to targeted disruption of the bacterial membrane and leakage of intracellular contents^11, 14^. This unique mechanism of action of AMPs does not require cells to be metabolically active and is not impaired by the dormant and quiescent state of bacteria in stationary-phase of growth^30^. The results obtained provide valuable insight into utilizing D-RR4 as a potential future therapeutic option for treatment of persistent bacterial infections.

### D-RR4 is capable of killing *P. aeruginosa* and *A. baumannii* harboring inside macrophages

**Figure 2:**
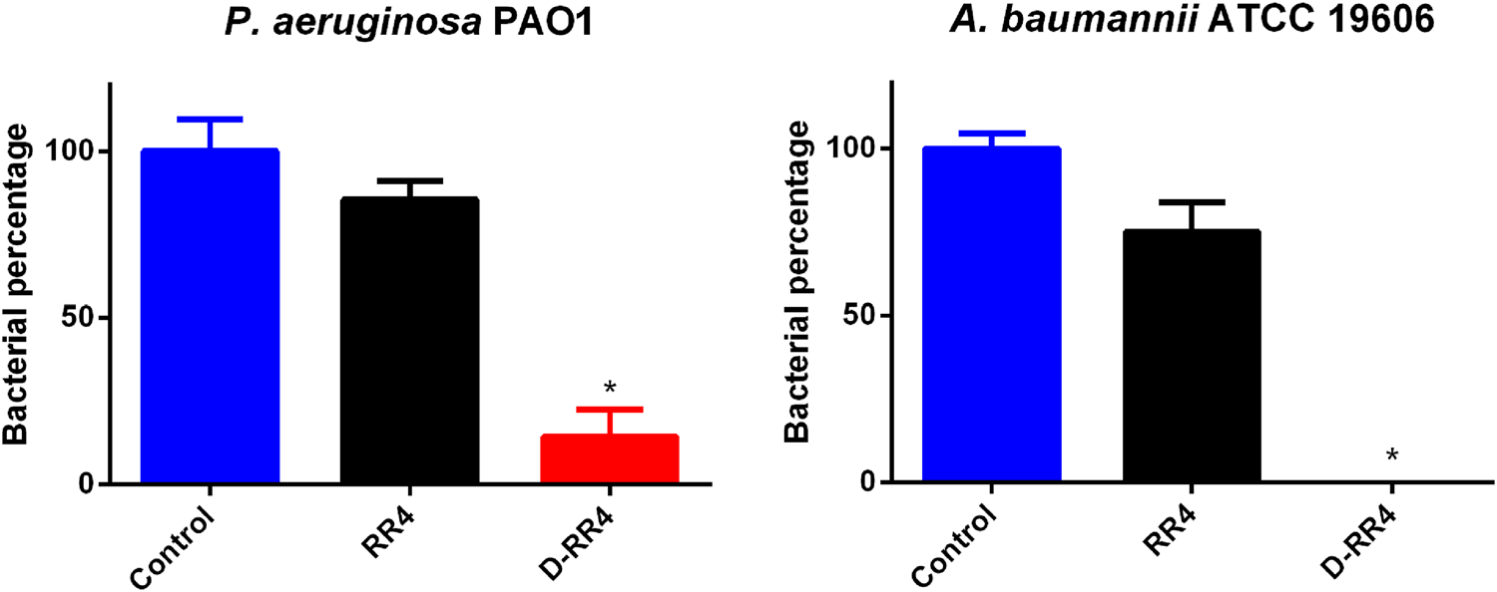
Intracellular antibacterial activity of peptides in infected murine macrophage cells (J774A.1). The effect of RR4 and D-RR4, at 8 × MIC, to kill intracellular *P. aeruginosa* PAO1 and *A. baumannii* ATCC 19606 inside infected J774A.1 cells after treatment for 24 hours. Statistical analysis was calculated using one-way ANOVA, with post hoc Tukey’s multiple comparisons test. *P* < 0.05 was considered significant. One asterisk (*) indicates significance from the negative control. Results are expressed as means from three biological replicates ± standard deviation.

*P. aeruginosa* and *A. baumannii* were considered to be primarily extracellular pathogens for many years. However, recent studies have revealed that these pathogens can gain entry and persist inside both phagocytic and non-phagocytic eukaryotic cells such as alveolar macrophages and lung epithelial cells^31–33^. Thus we investigated if RR4 and D-RR4 would be capable of killing intracellular *P. aeruginosa* and *A. baumannii* residing in murine macrophages. As demonstrated in Fig. 2, only the D isoform of RR4significantly reduced both intracellular *P. aeruginosa* and *A. baumannii*. D-RR4 (at 8 × MIC) killed 96.00% ± 1.15 and 100.00% ± 0.00 of intracellular *P. aeruginosa* and *A. baumannii*, respectively. In contrast, the L isoform of RR4 (at 8 × MIC) produced a 14.60% ± 5.50 and 25.00% ± 8.90 reduction of intracellular *P. aeruginosa* and *A. baumannii*. The enhanced efficacy of D-RR4 compared to RR4 may be related to its resistance to proteolysis by serum and other cellular enzymes, as described above^34^. For example, Cappiello *et al*. found that a diastereomer of the amphibian peptide Esc(1–21), Esc(1–21)-1c, had higher efficacy than its all L peptide in killing intracellular *P. aeruginosa* ^34^. The researchers found Esc(1–21)-1c was more resistant to bacterial and human elastase (compared to the all L peptide form)^34^. The potent intracellular activity of D-RR4 may be applicable for investigation of treatment of certain chronic diseases such as cystic fibrosis where *P. aeruginosa* persists inside eukaryotic cells as pods leading to treatment failure^32^.

### Mechanism of action of designed peptides

In order to gain insight into the mode of antibacterial mechanism of D-RR4, a series of experiments was conducted. First, damage to the bacterial outer membrane by D-RR4 was monitored by measuring the fluorescent intensity of *P. aeruginosa* PAO1 mixed with 1-N-phenylnaphthylamine (NPN) and then incubated with different concentrations of D-RR4. As demonstrated in Supplementary Fig. 4A, similar to the known membrane-targeting antimicrobial peptide LL-37, there was a significant and dose-dependent increase of fluorescence intensity of *P. aeruginosa* cultures treated with D-RR4. These findings indicate that D-RR4 likely targets the bacterial outer membrane leading to partitioning of NPN into the outer membrane and increased fluorescence.

Damage of the bacterial inner (cytoplasmic) membrane by peptides was monitored by measuring the fluorescence intensity of *P. aeruginosa* PAO1 combined with propidium iodide and then subsequently treated with D-RR4. As demonstrated in Supplementary Fig. 4B, the fluorescence intensity did not change in untreated bacteria confirming the cytoplasmic membrane remained intact. However, when D-RR4 and LL-37 were added to bacterial cultures, a rapid and significant increase in fluorescence was evident. At 10 × MIC, D-RR4 rapidly perturbed the inner membrane of bacteria more than D-RR4 tested at 5 × MIC. To confirm our results, we assayed the ATP level of bacterial cultures after exposure to D-RR4 compared to untreated bacteria. Physiologically, ATP is maintained inside bacterial cells with intact cell membranes. Leakage of ATP into the extracellular environment is indicative of membrane damage. As demonstrated in Supplementary Fig. 4C, similar to LL-37, there was a significant increase in luminescent intensity when *P. aeruginosa* PAO1 cells were treated with D-RR4 compared to the untreated control.

To further confirm the membrane-damaging effect of D-RR4 on bacteria, transmission electron microscopy (TEM) was conducted on thin sections of *P. aeruginosa* PAO1 treated with two different concentrations of D-RR4 (Supplementary Fig. 5). TEM micrographs of untreated bacteria revealed cell membranes that were intact and had a distinct cell wall (Supplementary Fig. 5A and 5B). The cytoplasmic region displayed a homogenous electron density. However, after exposure to D-RR4, noticeable damage to the bacterial cell was observed. Bacteria treated with a low concentration of D-RR4 (1 × MIC) exhibited disintegrated membranes with blebs and an increase in the periplasmic space (Supplementary Fig. 5C and D). Mesosomes, invaginations and condensed materials with similar electron density to that of the cell wall murein layer were also observed under membranes (Supplementary Fig. 5C and D). Bacteria treated with a higher concentration of D-RR4 (10 × MIC) exhibited more pronounced damage to the cell membranes as complete loss of membrane integrity and the cell wall were clearly observed (Supplementary Fig. 5E,F and G). Leakage of cytoplasmic contents that resulted in heterogeneous cytoplasmic densities, were also observed (Supplementary Fig. 5E and G). Ghost cells due to complete loss of cytoplasmic contents were also evident at higher peptide concentrations (Supplementary Fig. 5H). Collectively, these data confirm the membrane-damaging effect of D-RR4 against *A. baumannii* and *P. aeruginosa*.

### D-RR4 is capable of neutralizing bacterial lipopolysaccharide (LPS)

**Figure 3:**
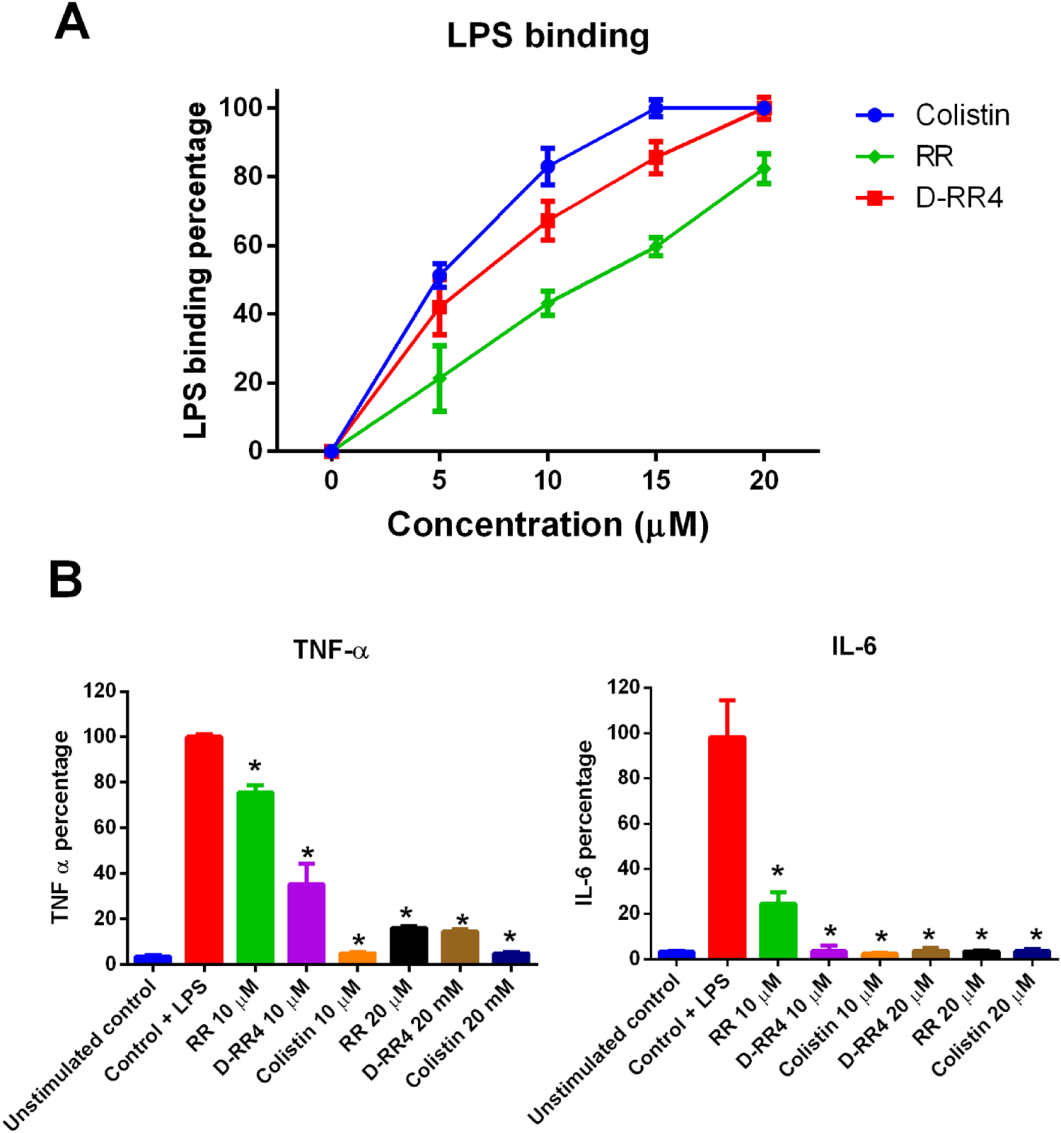
Ability of peptides to neutralize LPS and reduce inflammatory cytokines expression. (**A**) LPS binding activity of peptides. Peptides at different concentrations were incubated with one endotoxin unit (EU) of LPS at 37 °C for 30 minutes. Colistin was used as a positive control due to its high binding affinity for LPS. The binding of peptides with LPS is expressed as percent change relative to the untreated samples. (**B**) Anti-inflammatory effect of peptides on LPS-stimulated macrophages. J774A.1 cells were stimulated with LPS (150 ng/mL final concentration) in the presence of different concentrations of peptides. Cells stimulated with LPS alone and untreated cells served as controls. Cells were incubated for six hours at 37 °C before the supernatant from each treatment group was collected. Detection of tumor necrosis factor-α (TNF-α) and interleukin-6 (IL-6) in supernatants was determined using ELISA. Cytokine levels are expressed as percent change relative to the LPS-stimulated control. Statistical analysis was calculated using one-way ANOVA, with post hoc Tukey’s multiple comparisons test. *P* < 0.05 was considered significant. One asterisk (*) indicates significant difference with the negative control.

We investigated if our peptides could bind to and neutralize the effect of LPS. As presented in Fig. 3, RR and its analog, D-RR4 were able to bind to LPS *in vitro* as evident by inhibition of the LPS-induced activation of the LAL enzyme (*Limulus* amoebocyte lysate). RR produced a concentration-dependent inhibition of the LAL enzyme of 21.3%, 43.2%, 59.6% and 82.4% at 5, 10, 15 and 20 µM peptide concentration, respectively. D-RR4 demonstrated a more significant inhibition than RR, producing a 42.0%, 67.3%, and 85.6% reduction at 5, 10 and 15 µM, respectively. Complete inhibition was achieved at 20 µM. We suspect the high cationic charge of D-RR4, in relation to RR, is responsible for the enhanced binding affinity of this peptide to LPS. Colistin, a known LPS binding agent, showed significant inhibition generating a 51.2% and 83.0% reduction at 5 and 10 µM. Complete inhibition of LPS-induced activation of the LAL enzyme was achieved by colistin at both 15 and 20 µM (Fig. 3A).

Next, we examined if RR and D-RR4 could reduce the endotoxin-induced proinflammatory cytokine response in murine macrophages using ELISA. As depicted in Fig. 3B, RR and its analog, D-RR4 were able to inhibit LPS-induced proinflammatory cytokine production in macrophages, in a manner similar to colistin. The inhibition of cytokine production was found to be concentration-dependent. RR, at 10 µM, decreased TNF-α and IL-6 levels by 24.4% and 75.4%, respectively. At 20 µM, RR inhibited 84.0% and 96.5% of TNF-α and IL-6 production, respectively. At 10 µM, D-RR4 was superior to RR as it decreased TNF-α and IL-6 production by 64.7% and 96.4%, respectively. At 20 µM, D-RR4 inhibited 85.6% and 96.5% of TNF-α and IL-6 production, respectively. Colistin, as expected, produced a > 95% inhibition of both cytokines at the tested concentration (10 and 20 µM) (Fig. 3B). The capability of the designed peptides to reduce endotoxin-mediated proinflammatory cytokine production provides a potential avenue for their development as antibacterial agents alone or as an adjunctive with antibiotics to treat sepsis.

### *In vivo* efficacy of peptides in a *C. elegans* infection model

**Figure 4:**
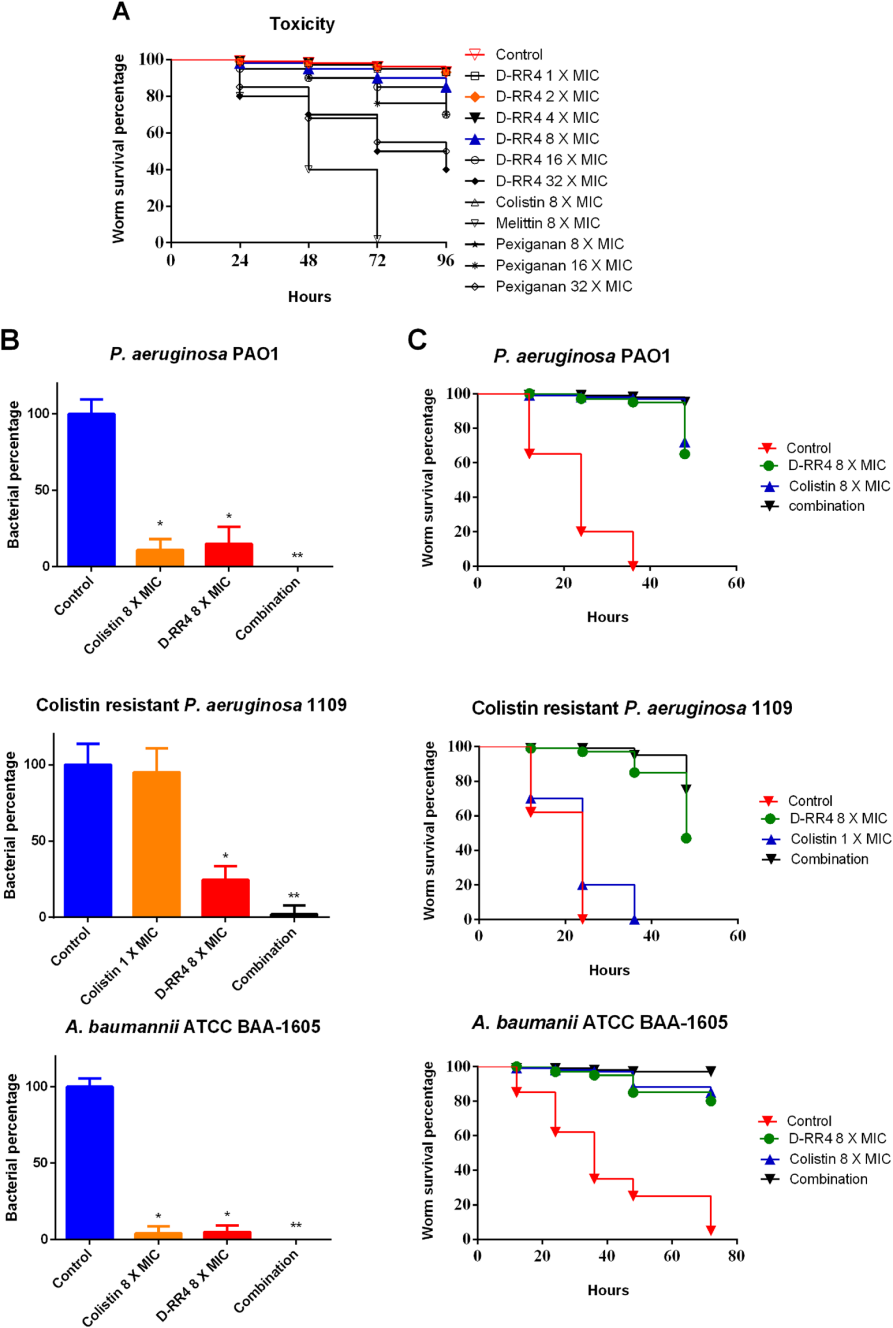
Antibacterial activity of D-RR4 and colistin in *C. elegans* models of bacterial infection. (**A**) Evaluation of toxicity of peptides in *Caenorhabditis elegans*. *C. elegans* strain glp-4;sek 1 was grown for four days in the presence of peptides at different concentrations. Worms were monitored daily and the live worms were counted. Results are expressed as a Kaplan-Meier survival curve. (**B**) Evaluation of antimicrobial activity of peptides to reduce the bacterial CFU inside infected *Caenorhabditis elegans*. *C. elegans* strain glp-4; sek 1 were infected with *P. aeruginosa* PAO1, colistin-resistant *P. aeruginosa* 1109, or *A. baumannii* ATCC BAA-1605. After infection, worms were treated with D-RR4 (at 8 × MIC), colistin (at 8 × MIC for all strains except colistin-resistant *P. aeruginosa* 1109 (tested at 1 × MIC)), or a combination of both agents. After treatment, worms were lysed and bacteria were counted. One asterisk (*) indicates significant difference with the negative control. Two asterisks (**) indicates significant difference with the monotherapy treatment (*P* < 0.05). Results are expressed as means from three biological replicates ± standard deviation. (**C**) Efficacy of peptides in a *Caenorhabditis elegans* model of bacterial infection. *C. elegans* strain glp-4; sek 1 were infected with *P. aeruginosa* PAO1, colistin resistant *P. aeruginosa* 1109, or *A. baumannii* ATCC BAA-1605. After infection, worms were treated with D-RR4 (at 8 × MIC), colistin (at 8 × MIC for all strains except colistin-resistant *P. aeruginosa* 1109 (tested at 1 × MIC)), or a combination of both. Worms were monitored daily and the live worms were counted. Results are expressed as a Kaplan-Meier survival curve. *C. elegans* receiving no treatment served as a negative control.

To test the efficacy of the synthetic peptides to protect against infections *in vivo*, we utilized a *C. elegans* model of bacterial infection. This model has been used extensively for early-stage evaluation of promising antimicrobials^18, 35, 36^. Initially, the lethal dose of D-RR4 against uninfected nematodes was determined. D-RR4 and colistin were not toxic to worms at 8 × MIC (Fig. 4A). However, melittin completely killed *C. elegans* after 3 days.

We next evaluated the ability of D-RR4 to reduce bacterial burden (CFU) inside the gut of infected worms. As depicted in Fig. 4B, D-RR4 produced a significant reduction of *P. aeruginosa* at 8 × MIC (85%). Similarly, colistin (8 × MIC) produced an 89% reduction in bacterial load. Complete clearance of bacteria from worms was achieved when D-RR4 was combined with colistin. Next, we evaluated D-RR4’s and colistin’s activity against a colistin-resistant *P. aeruginosa* 1109 strain isolated from a CF patient. Colistin was unable to significantly decrease bacterial burden, in contrast to D-RR4, which produced a 75% reduction. Interestingly, the combination of D-RR4 and colistin showed enhanced killing as the combination reduced 98% of colistin-resistant *P. aeruginosa* in infected worms (Fig. 4B). When the same test agents were evaluated against *A. baumannii*, a similar pattern was observed. D-RR4 and colistin independently decreased bacterial burden by 95% and 96%. When the two agents were tested in combination, complete eradication of bacterial load was observed (Fig. 4B).

Bolstered by these results, we next examined whether D-RR4 could protect infected worms from the cidal activity of bacteria. Interestingly, *P. aeruginosa* PAO1 killed 80% of untreated worms after 24 hours and 100% mortality was observed after 36 hours (Fig. 4C). D-RR4 and colistin significantly increased the rate of survival for *Pseudomonas*-infected *C. elegans* in contrast to untreated worms, giving nearly complete protection for 24 and 36 hours. A 65% to 72% protection was observed up to 48 hours. A combination of D-RR4 and colistin showed complete protection of worms, even up to 48 hours (Fig. 4C). The survival experiment with *C. elegans* was repeated to examine the impact of D-RR4 to enhance survival of worms infected with colistin-resistant *P. aeruginosa* 1109. As expected, this strain demonstrated higher virulence than PAO1 as it completely killed *C. elegans* after 24 hours. While colistin failed to protect worms from death, D-RR4 significantly increased the survival rate of *C. elegans* and gave nearly complete protection after 24 hours. After 36 and 48 hours respectively, 85% and 47% of worms treated with D-RR4 remained alive. Interestingly, a combination of D-RR4 and colistin showed enhanced protection as all worms remained alive after 36 hours. Furthermore, 75% of infected worms remained alive after 48 hours (Fig. 4C).

Next, we assessed the ability of D-RR4 to prolong survival of worms infected with *A. baumannii*. Interestingly, *A. baumannii* BAA-1605 killed worms more slowly than *P. aeruginosa*. In the untreated control group, 75% of worms were dead after 48 hours while 95% were dead after 72 hours. A notable protective effect was provided for infected worms treated with either D-RR4 or colistin. All infected worms treated with D-RR4 or colistin remained alive 36 hours while 80–85% of worms were alive after 72 hours. Consistent with the *in vitro* data, the combination of D-RR4 and colistin completely protected infected worms from death up to 72 hours (Fig. 4C).

In conclusion, we successfully designed modified derivatives of a short synthetic peptide, RR, to enhance its potency and spectrum of activity against important Gram-negative pathogens. RR4 and its D- enantiomer, D-RR4, were found to be the most potent analogues of RR D-RR4 exhibited improved antibacterial activity against both multidrug-resistant *P. aeruginosa* and *A. baumannii*. Furthermore, D-RR4’s antibacterial effect was not lost in the presence of physiologically-relevant concentrations of salts, acidic pH, human serum, and both bacterial and mammalian proteases. Additionally, D-RR4 demonstrated the ability to disrupt mature *P. aeruginosa* biofilm and kill intracellular *P. aeruginosa* harboring inside macrophages. Furthermore, the antibacterial activity of D-RR4 was validated in *C. elegans* models of *P. aeruginosa* and *A. baumannii* infection. Thus, we posit that D-RR4 does have promise as a novel therapeutic option for *P. aeruginosa* and *A. baumannii* infections though further work is necessary to successfully translate D-RR4 to the clinic.

## Methods

### Bacterial isolates, peptides and reagents

Clinical isolates of *P. aeruginosa* and *A. baumannii* are presented in Supplementary Tables S1, S2 and S3. All experiments were carried out in accordance with relevant guidelines and regulations and were approved by the Institutional Biosafety Committee of Purdue University. Peptides were synthesized using solid-phase 9-fluorenylmethoxy carbonyl (Fmoc) chemistry (GenScript, Piscataway, NJ). Melittin was purchased from Sigma-Aldrich (St. Louis, MO). Antibiotics were purchased from commercial vendors.

### Determination of minimum inhibitory concentration (MIC) values

The broth microdilution technique was utilized to assess the MIC of designed peptides according to the guidelines of the Clinical and Laboratory Standards Institute (CLSI)^37^.

### Circular dichroism spectroscopy (CD)

Data were recorded on a Jasco circular dichroism spectropolarimeter (Model J810) as described previously^11, 14^.

### Resistance to proteolytic digestion by mammalian and bacterial proteases

To examine the ability of peptides to resist proteolytic digestion by mammalian and bacterial proteases, RR4 and D-RR4 were incubated with trypsin or proteinase K at a molar ratio of 500: 1 (peptide: enzyme), respectively, in digestion buffer (50 mM Tris–HCl, pH 7.4) at 37 °C for four hours, as described previously^38^.

### Resistance of peptides to salts, plasma and serum

The activity of the designed peptides in different environmental conditions including salts, serum and plasma, was evaluated using the broth microdilution assay described above. *P. aeruginosa* PAO1 was treated with peptides under specific physiological conditions presented in Table 4.

### Toxicity of peptides against mammalian cell lines

The peptides were assayed for potential *in vitro* toxicity against human keratinocytes (HaCaT) and murine macrophage cells (J774A.1), as described before^39^, with the following modifications. Cells were treated with peptides at different concentrations for 24 hours. The therapeutic index of the peptides was calculated as the ratio of mammalian EC_50_ (the concentration of peptide that inhibited growth of 50% of the mammalian cells) to the MIC_50_ of peptides against bacteria. When there was no detectable cytotoxicity at 256 µM, a higher concentration (512 µM) was used to calculate the therapeutic index. Larger therapeutic index values indicate greater cell selectivity of the peptides^40^.

### Hemolysis assay

Peptides were assayed for hemolytic activity against human red blood cells, as described previously^7^.

### Efficacy of peptides on bacterial biofilms

To examine the peptides’ ability to disrupt bacterial biofilms, the microtiter dish biofilm formation assay was employed^41–43^.

### Time-kill assay against logarithmic and stationary phase bacteria

*P. aeruginosa* PAO1 and *A. baumannii* ATCC BAA-1605 were used for time kill assay as described before^44, 45^.

### Mechanism of action studies

#### Outer membrane permeabilization assay

Permeation of the bacterial outer membrane by peptides was assessed using the non-polar fluorescent dye, 1-N-phenylnaphthylamine (NPN), as described elsewhere^46^.

#### Inner (cytoplasmic) membrane permeabilization assay

Damage of the bacterial inner (cytoplasmic) membrane after exposure to peptides was evaluated using propidium iodide, as described elsewhere^47^.

#### ATP leakage assay

ATP leakage, from bacteria treated with peptides, was detected using a luminescence assay following the manufacturer’s (Promega) instructions.

#### Transmission electron microscopy

*P. aeruginosa* PAO1 was treated and fixed and examined with a Philips CM-100 microscope, as described previously^39^.

### Intracellular antibacterial efficacy of peptides in treating infected murine macrophages

In order to assess the peptides’ ability to kill intracellular bacteria, murine macrophages (J774A.1) were infected, as described in previous studies^31–33, 42, 48–51^. Briefly, J774A.1 cells were seeded and incubated as described above for the toxicity assessment. Following incubation, cells were infected with either *P. aeruginosa* PAO1 or *A. baumannii* ATCC 19606 (multiplicity of infection 100:1) in DMEM + 10% FBS for two hours. After infection, the wells were washed with 200 µL of media containing gentamicin (50 µg/ml) and further incubated for 30 minutes with gentamicin to kill extracellular bacteria. Drugs (using triplicate samples) were diluted in DMEM + 10% FBS (to a concentration equal to 8 × MIC) and added to appropriate wells. Medium alone was used as a negative control. After incubation, the media was aspirated and washed twice with PBS to remove any residual test agent. Next, 100 µL of PBS containing 0.01% triton X-100 was added to lyse the J774 cells. Subsequently, bacteria were diluted and plated on TSA plates. Plates were incubated at 37 °C for 16 hours. After incubation, bacteria were enumerated and analyzed. The experiment was repeated twice. The average percent reduction of bacterial growth for each treatment regimen has been reported.

## LPS studies

### Binding of peptides to lipopolysaccharide (LAL assay)

To determine the peptides’ ability to bind to LPS, a *Limulus* amoebocyte lysate (LAL) kit (Genscript, USA Inc.) was utilized, as described in previous reports^52^. The peptides were dissolved in pyrogen-free water and serially diluted in the same solvent. The peptides were then incubated with one endotoxin unit (EU) of LPS at 37 °C for 30 minutes in order to permit the peptides to bind LPS. The reaction mixture (50 μL) was added to 50 μL of LAL reagent, and further incubated for 10 minutes. Next, 100 μL of LAL chromogenic substrate was added. The mixture was incubated at 37 °C for six minutes. The optical density (at 545 nm) was determined using a microplate reader. Colistin was used as a positive control. The binding of peptides with LPS was expressed as the percent change relative to the untreated control.

### Anti-inflammatory effect of peptides on LPS-stimulated macrophages

To assess the anti-inflammatory effect of our peptides on LPS-stimulated macrophages, J774A.1 cells were seeded and incubated as described above (for toxicity assessment). Next, the cells were stimulated with LPS (150 ng/ml final concentration) in the presence of different concentrations of peptides (RR and D-RR4). Cells stimulated with LPS alone and untreated cells served as controls. Cells were incubated for six hours at 37 °C and the supernatant from each treatment was collected and stored at −20 °C until use. Tumor necrosis factor-α (TNF-α) and interleukin-6 (IL-6) present in the supernatant was quantified using ELISA, as described previously^8^. Cytokine levels were presented as the percent change relative to the LPS-stimulated control, using triplicate samples for each treatment condition.

### *In vivo* efficacy of peptides in a *C. elegans* infection model

Infection and treatment of *C. elegans* was done, as described previously, with the following modifications^51, 53, 54^. Briefly, synchronized adult worms were transferred to modified NGM (0.35% peptone) agar plates seeded with a lawn of *P. aeruginosa* PAO1, colistin-resistant *P. aeruginosa* PAO1, or *A. baumannii* ATCC BAA-1605, for infection. After infection, worms were collected and washed with PBS three times. Worms (~30 per sample) were transferred either to microcentrifuge tubes (for survival assessment) or 96-well plates (for bacterial CFU determination). D-RR4 and colistin (at 8 × MIC) were added, in triplicate, to all wells with one exception. Worms infected with colistin-resistant *P. aeruginosa* 1109 were treated with colistin at 1 × MIC (equal to 128 µM) in. Untreated worms served as a negative control. To assess the survival of infected *C. elegans*, worms were assessed for viability (live worms are sinusoidal with movement, whereas dead worms are rigid rods). In order to assess the bacterial load in worms, worms were first washed three times with PBS. They were subsequently lysed by addition of 200 mg of 1.0-mm silicon carbide particles (Biospec Products, Bartlesville, OK) to each tube and vortexing for one minute. Bacteria were plated onto TSA plates containing 100 µg/ml ampicillin to permit the selective growth of *P. aeruginosa* and *A. baumannii* over *Escherichia coli* OP50.

### Statistical analyses

Statistical analyses were performed using GraphPad Prism 6.0 software (GraphPad Software, La Jolla, CA, USA). Comparison between two groups was analyzed using a two-tailed unpaired Student’s t-test. Comparison between three or more groups was analyzed using one-way ANOVA, with post hoc Tukey’s multiple comparisons test. *P*-values of < 0.05 were considered significant.

## Electronic supplementary material

Supplementary materials
