# Supplementary material for: A short D-enantiomeric antimicrobial peptide with potent immunomodulatory and antibiofilm activity against multidrug-resistant Pseudomonas aeruginosa and Acinetobacter baumannii
Source: Sci Rep. 2017 Jul 31;7:6953. doi: 10.1038/s41598-017-07440-0 (PMC5537347; doi:10.1038/s41598-017-07440-0)
Supplement: Supplementary file 1 — Supplementary materials [file 41598_2017_7440_MOESM1_ESM.pdf]

Supplementary materials

A short D-enantiomeric antimicrobial peptide with potent immunomodulatory and antibiofilm activity against multidrug-resistant *Pseudomonas aeruginosa* and *Acinetobacter baumannii*

Mohamed F. Mohamed[1,], Anna Brezden[2], Haroon Mohammad[1], Jean Chmielewski[2,3], Mohamed N. Seleem[1,3]*

[1]Department of Comparative Pathobiology, Purdue University, West Lafayette, IN, 47907, USA
[2]Department of Chemistry, Purdue University, West Lafayette, IN 47907, (USA)
[3]Purdue Institute for Inflammation, Immunology, and Infectious Disease, Purdue University, West Lafayette, IN 47907, USA.

Supplementary Table S1: *Pseudomonas aeruginosa* isolates used in the study.

	Strain	Phenotype
1	*P. aeruginosa* PAO1	Biofilm-producing strain, quality control strain.
2	*P. aeruginosa* ATCC 9027	Quality control strain, produces rhamnolipid surfactant, isolated from an outer ear infection.
3	*P. aeruginosa* ATCC 15442	Quality control strain for testing antimicrobial agents.
4	*P. aeruginosa* ATCC 25619	Quality control strain.
5	*P.s aeruginosa* ATCC 27853	Isolated from blood sample, quality control strain.
6	*P. aeruginosa* ATCC 9721	Bacterial resistance testing adhesives, produces lipases active at pH 5.5 and 7.5, quality control strain
7	*P. aeruginosa* ATCC 10145	Produces lipases active at pH 5.5 and 7.5, quality control strain.
8	*P.s aeruginosa* ATCC BAA-1744	Clinical isolate, quality control strain.
9	*P. aeruginosa* ATCC 35032	Quality control strain.

Supplementary Table S2: Clinical isolates of colistin-resistant *P. aeruginosa* isolated from cystic fibrosis patients.

	Strain	Phenotype
1	*P. aeruginosa* 1603	Isolated from patient in Copenhagen, Denmark, 2003.
2	*P. aeruginosa* 1131	Isolated from patient in Leeds, UK, 1999.
3	*P. aeruginosa* 1017	Isolated from patient in Aarhus, Denmark, 1996.
4	*P. aeruginosa* 1571	Isolated from patient in Copenhagen, Denmark, 2002/1995.
5	*P. aeruginosa* 1015	Isolated from patient in Copenhagen, Denmark, 1996.

6	*P. aeruginosa* 1016	Isolated from patient in Aarhus, Denmark, 1996/1985.
7	*P. aeruginosa* 1109	Isolated from patient in the UK, 1990s.
8	*P. aeruginosa* 1020	Isolated from patient in Copenhagen, Denmark, 1998/1995.
9	*P. aeruginosa* 1611	Isolated from patient in Copenhagen, Denmark, 2001.
10	*P. aeruginosa* 1125	Isolated from patient in Leeds, UK, 1997.
11	*P. aeruginosa* 1133	Isolated from patient in Leeds, UK, 2000.

Supplementary Table S3: Clinical isolates of *Acinetobacter baumannii* used in the study.

	Strain	Phenotype
1	*A. baumannii* ATCC BAA-1605	Isolated from sputum of military personnel returning from Afghanistan entering a Canadian hospital, June 30, 2006. A multidrug-resistant strain resistant to ceftazidime, gentamicin, ticarcillin, piperacillin, aztreonam, cefepime, ciprofloxacin, imipenem, and meropemem.<br>Sensitive to Amikacin and Tobramycin.
2	*A. baumannii* ATCC BAA-747	Human clinical specimen isolated from ear pus. Quality control strain.
3	*A. baumannii* ATCC 19606	Quality control strain.
4	*A. baumannii* -- H72721 (NR-9667)	Isolated at Landstahl Regional Medical Center in Germany in June 2006 from the sputum of a Canadian soldier injured in Afghanistan.
5	*A. baumannii* -- Isolate 1 (NR-13374)	Isolate 1 was obtained from a human tracheal aspirate in 2008.
6	*A. baumannii* -- Isolate 2 (NR-13375)	Obtained from human sputum in 2008.
7	*A. baumannii* -- Isolate 9 (NR-13382)	Obtained from human blood in 2008.
8	*A. baumannii* -- 3-137 (OIFC137) (NR-17777)	Isolated in May 2003 from a catheter tip of a human subject at Walter Reed Army Medical Center, Washington, D.C., USA (multidrug-resistant strain).
9	*A. baumannii* -- 5-032 (OIFC032) (NR-17778)	A human isolate collected in May 2003 from the wound of a patient at the Landstuhl Regional Medical Center in Landstuhl, Germany.

10	*A. baumannii* -- OIFC109 (NR-17780)	A human isolate collected in June 2006 from the residual limb wound of a patient at the Walter Reed Army Medical Center, Washington, D.C., USA.
11	*A. baumannii* -- BC-5 (NR-17783)	Isolated in 2007 from a nosocomial spread of war-related multidrug-resistant *A. baumannii* in a civilian hospital in British Columbia, Canada. The infection originated from a soldier who was evacuated from Landstuhl Regional Medical Center in Landstuhl, Germany (multidrug-resistant isolate).
12	*A. baumannii* -- Naval-17 (NR-17784)	A human isolate collected in June 2006 from the wound of a patient at the National Naval Medical Center in Bethesda, Maryland, USA.
13	*A. baumannii* -- Naval-18 (NR-17785)	A human isolate collected in June 2006 from the wound of a patient at the National Naval Medical Center in Bethesda, Maryland, USA.
14	*A. baumannii* -- Naval-81 (NR-17786)	Isolated on October 9, 2006, from human blood at the National Naval Medical Center in Bethesda, MD, USA.
15	*A. baumannii* -- WC-136 (NR-19298)	Isolated in January 2008 from healthcare equipment surface at Camp Delta in Iraq (multidrug-resistant isolate).
16	*A. baumannii* -- WC-487 (NR-19299)	Isolated in March 2008 from an intact human skin surface at Camp Delta in Iraq (multidrug-resistant isolate).

Supplementary Table S4: Toxicity and therapeutic index of designed peptides.

Peptide	MIC_{50} (μM)[a]	EC_{50} (μM)[b] J774.1/ HaCaT	TI[c] J774.1/ HaCaT	HC_{50}[d] (μM)	TI[c] RBCs
RR	64	128/256	2/4	>256	8
RR1	>256	>256/>256	NA[e]	>256	NA
RR2	4	128/64	32/16	256	64
RR3	8	128/128	16/16	>256	64
RR4	4	64/128	16/32	256	64
D-RR4	2	64/64	32/32	256	128
Melittin	8	2/4	0.25/0.5	4	0.5

[a] MIC_{50} (μM) of peptide against *P. aeruginosa* and *A. baumannii* isolates (Table 2).

[b] EC_{50} is the peptide concentration that inhibited 50% of the macrophage cell line (J774.1) or human keratinocyte (HaCaT) cell line viability.

[c] Therapeutic index is the ratio of the EC_{50} or HC_{50} value over the MIC_{50} value.

[d] HC_{50} is the peptide concentration that resulted in 50% hemolysis of red blood cells.

[e] NA. not applicable.

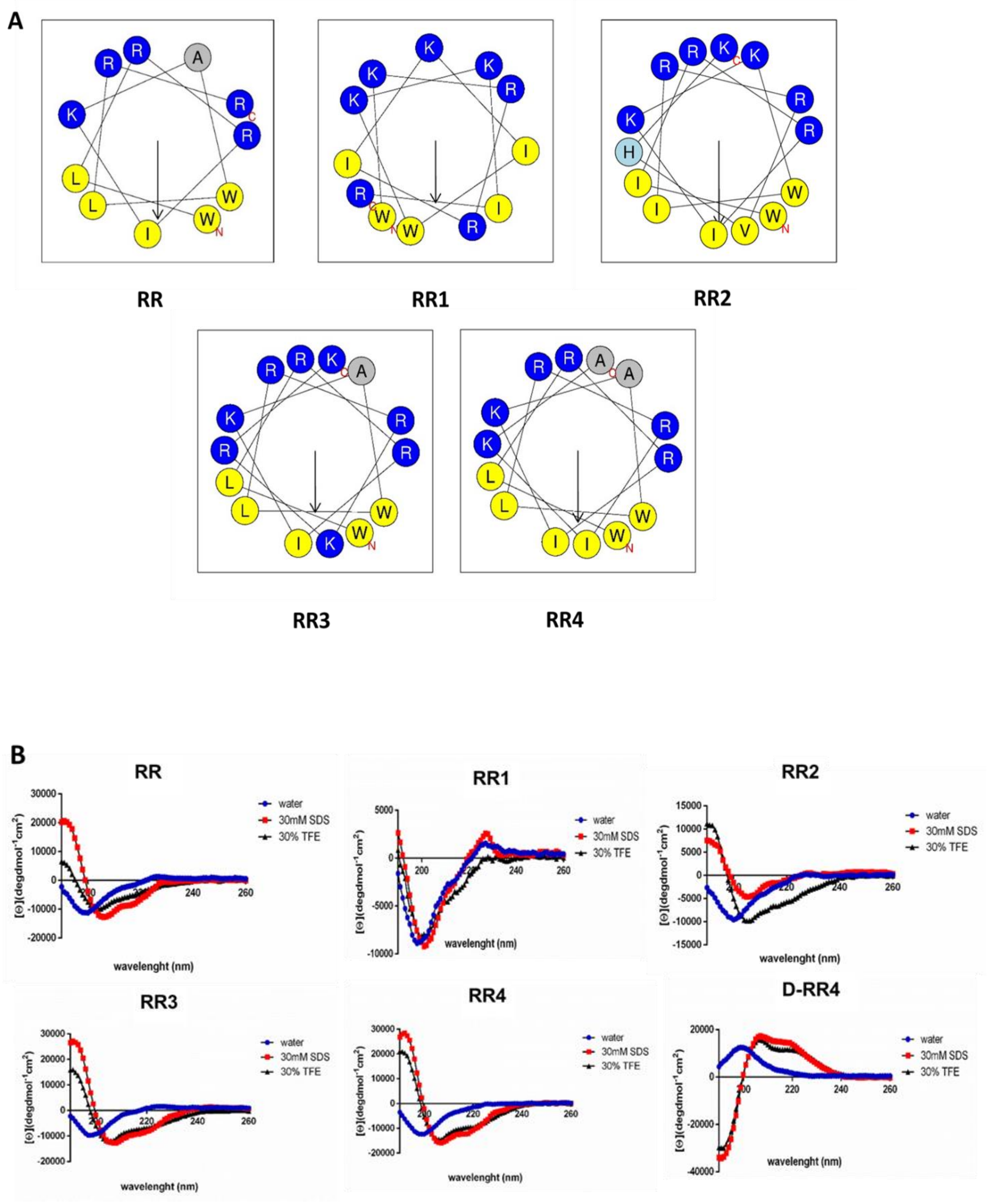

Supplementary Figure 1: A) Helical wheel projections of the designed peptides. Yellow and blue circles represent hydrophobic and hydrophilic residues, respectively. Gray and bright blue

circles represent alanine and histidine residues, respectively. The arrows indicate the directions and magnitudes of the hydrophobic moments (μH) determined for each peptide - N, amino terminus; C, carboxy terminus. (B) CD analyses of the designed peptides at 50 μM concentration in deionized water, 30 mM sodium dodecyl sulfate (SDS) or 30% trifluoroethanol (TFE).

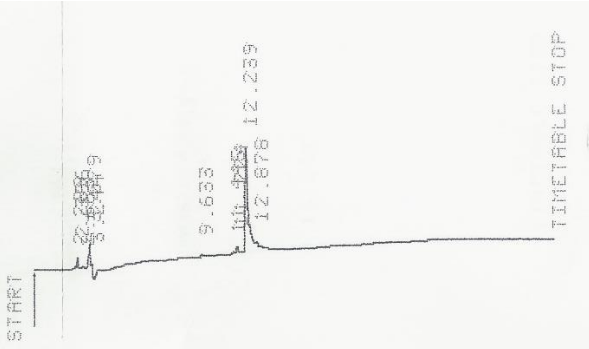

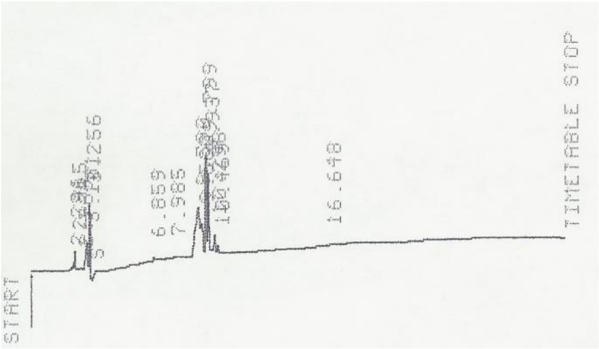

RR4 : Proteinase K Time 0

RR4 : Proteinase K Time 4 hours

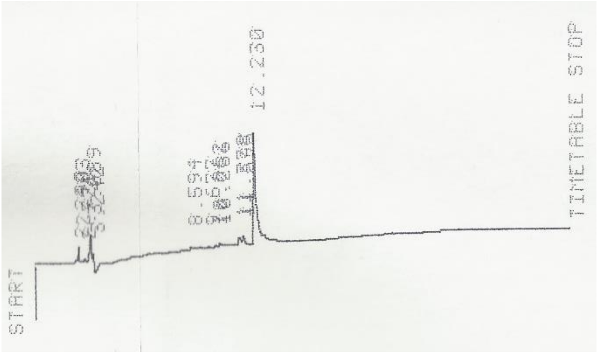

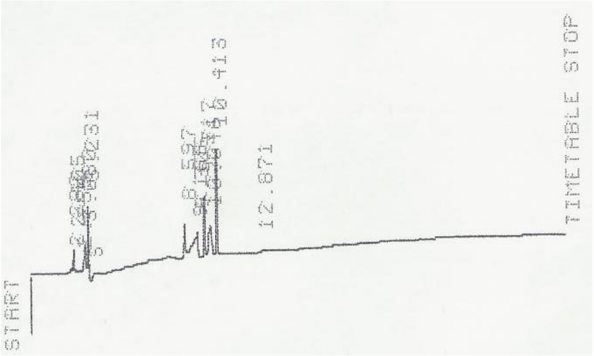

RR4 : Trypsin Time 0

RR4 : Trypsin Time 4 hours

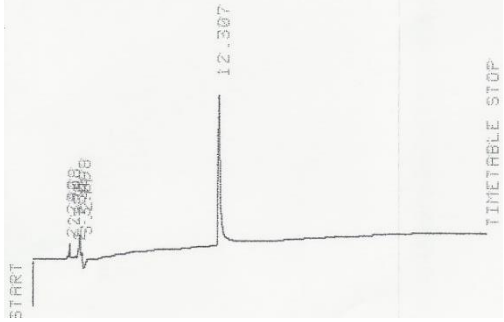

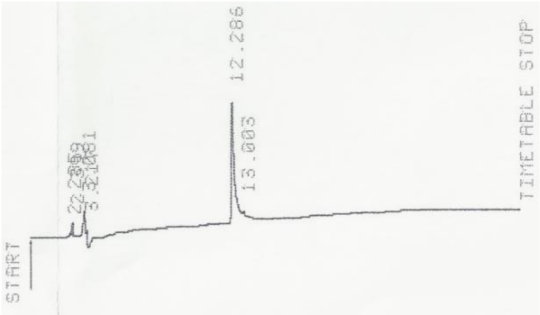

D-RR4 : Proteinase K Time 0

D-RR4 : Proteinase K Time 4 hours

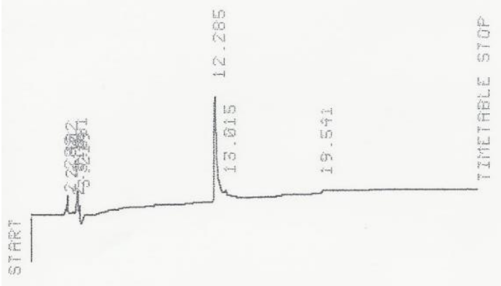

D-RR4 : Proteinase K Time 24 hours

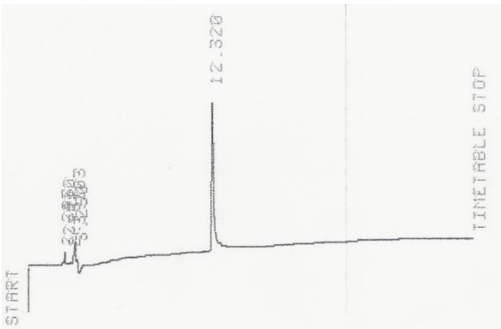

D-RR4 : Trypsin Time 0

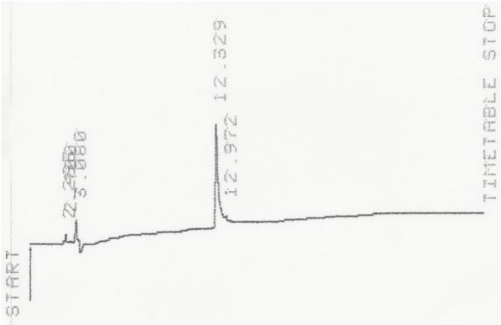

D-RR4: Trypsin Time 4 hours

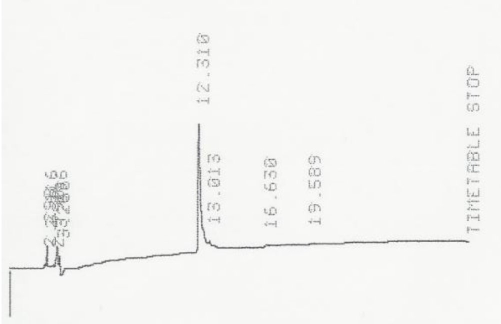

D-RR4 : Trypsin Time 24 hours

Supplementary Figure 2: Analytical HPLC spectra demonstrating proteolytic activity of trypsin and proteinase K on RR4 and D-RR4 after 0, 4 and 24 hours of incubation.

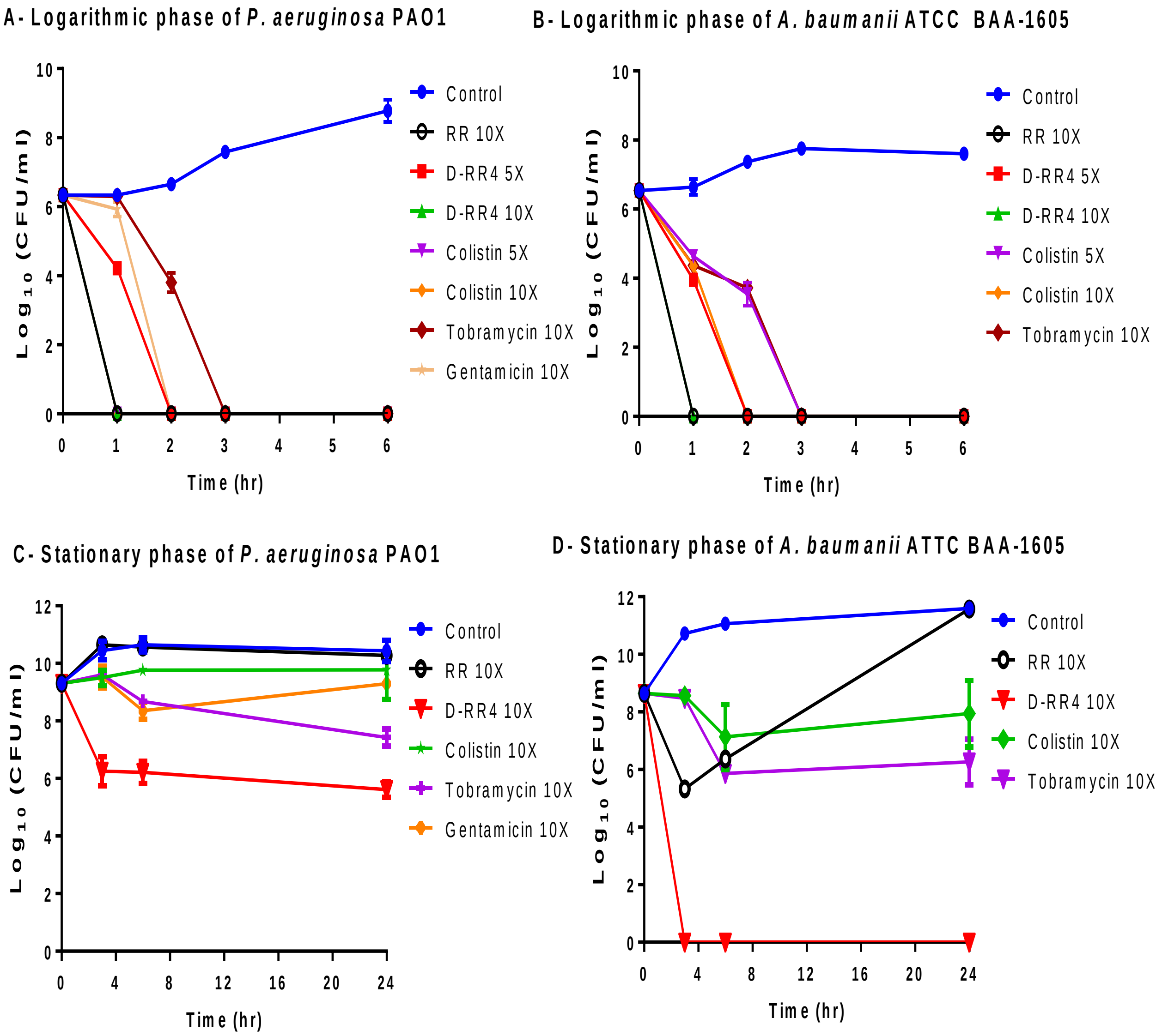

Supplementary Figure 3: The killing kinetics of peptides and antibiotics against logarithmic (A&B) and stationary phase (C&D) cultures of *P. aeruginosa* PAO1 (A&C) and *A. baumannii* ATCC BAA-1605 (B&D). **(A&B)** Bacteria in logarithmic-phase of growth were exposed to D-RR4 and colistin at 5 × and 10 × MIC or RR, tobramycin and gentamicin at 10 × MIC. **(C&D)** The killing kinetics of bacteria in stationary-phase of growth exposed to peptides and antibiotics at 10 × MIC. Untreated samples served as a negative control. The killing curves were identicalfor RR, D-RR4 and colistin at 10 × MIC in panel A and identical for RR and D-RR4 at 10 × MIC in panel B. Results are presented as means ± SD (n = 3); data without error bars indicate that the SD is too small to be seen.

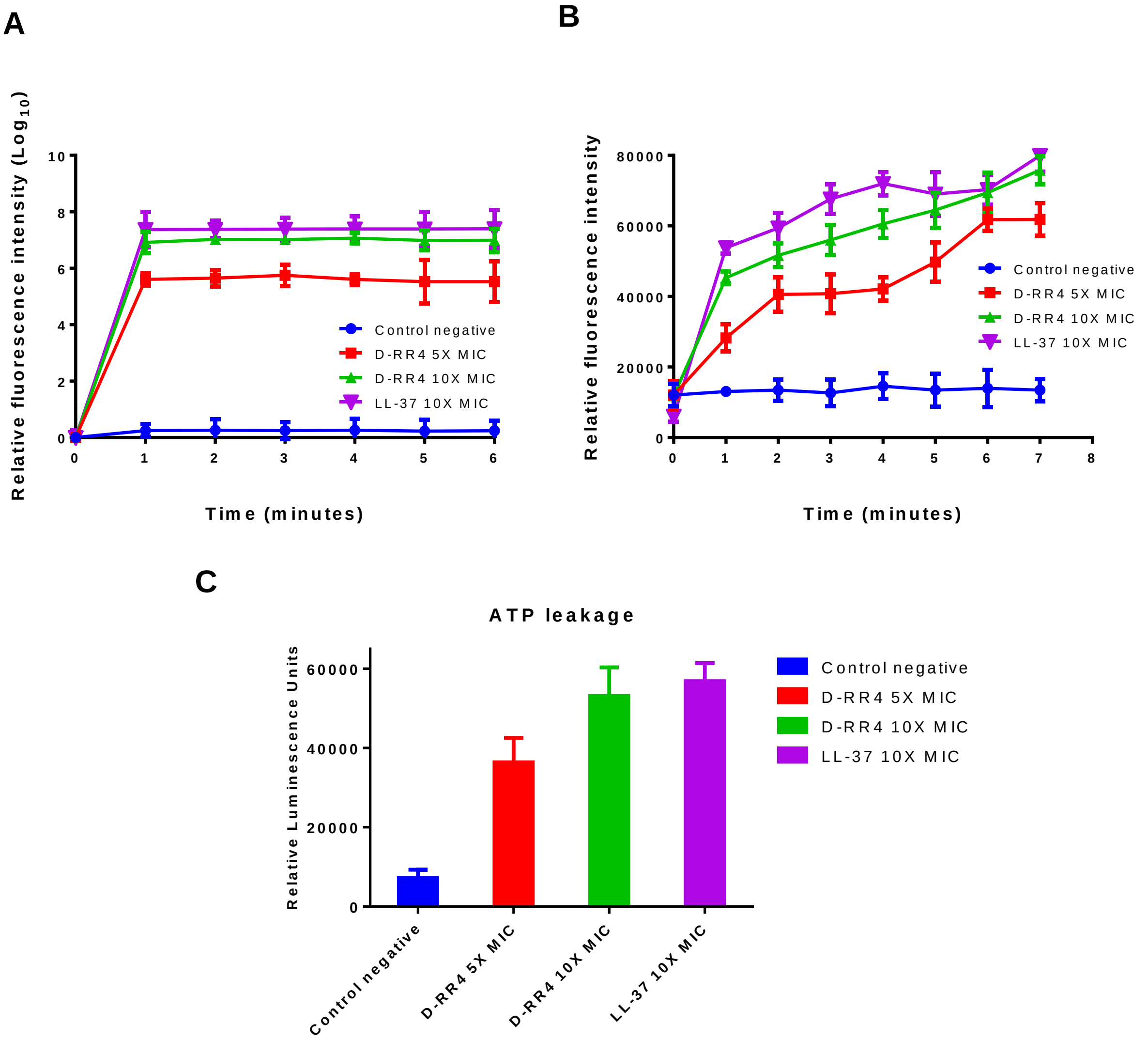

SupplementaryFfigure 4: A) Permeabilization of the outer membrane of *P. aeruginosa* PAO1 by antimicrobial peptides as indicated by the enhanced uptake of 1-N-phenylnaphthylamine (NPN). Fluorescence intensity, caused by the partitioning of NPN into the outer membrane, was monitored after addition of either D-RR4 or LL-37. Data are presented as means ± standard error of the means (n = 3). **B)** Permeabilization of the inner (cytoplasmic) membrane by D-RR4 and LL-37 is indicated by propidium iodide fluorescence. **C)** Release of ATP from bacteria treated with peptides was detected using a luminescence assay. Each experiment was performed in duplicate, and the values represent means ± standard deviation.

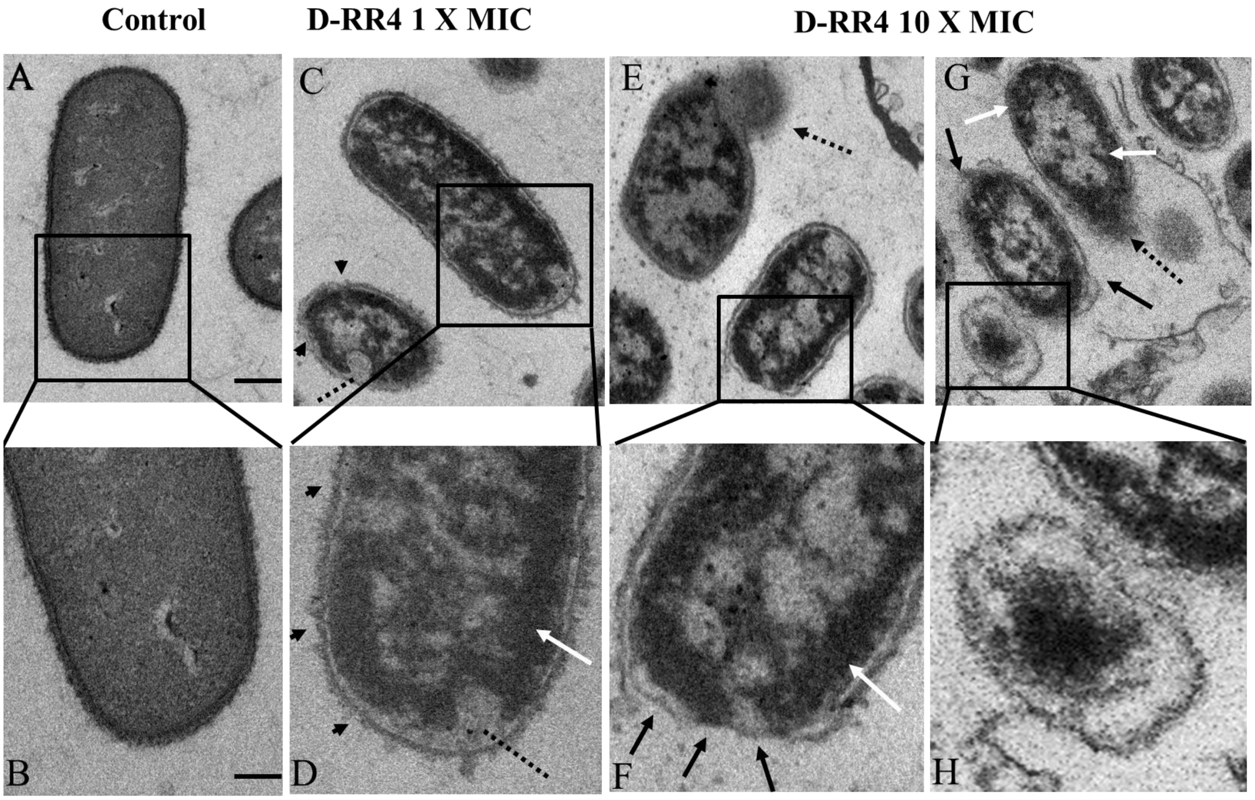

Supplementary Figure 5: Transmission Electron Microscopy micrographs of untreated and peptide-treated *P. aeruginosa* PAO1. The cells of untreated bacteria are intact, with a well-defined cell membrane (panels A & B). Bacteria treated with low concentration of D-RR4 (1 × MIC) showed disintegrated membranes with blebs and increases in periplasmic space (black arrow heads), mesosomes and invaginations (dashed lines), and condensed materials under membranes (white arrows) (panels C & D). Bacteria treated with a higher concentration of D-RR4 peptide (10 × MIC) (panels E-H) showed complete loss of membranes (black arrows) and leakage of cytoplasmic contents (dashed arrow). Ghost cells due to loss of most cytoplasmic contents were also evident at higher peptide concentrations (panel H). The lower panel is a higher magnification of the region highlighted by a square in the upper panel. Upper bar, 500 nM; lower bar 100 nM.
